# Hemorrhagic Septicæmia in Cattle

**Published:** 1900-12

**Authors:** S. D. Brimhall, Louis B. Wilson

**Affiliations:** Veterinarian; First Assistant Bacteriologist


### HEMORRHAGIC SEPTICÆMIA IN CATTLE.

### REPORT FROM MINNESOTA STATE BOARD OF HEALTH.[2]

S. D. BRIMHALL, V.M.D., VETERINARIAN.

LOUIS B. WILSON, M.D., FIRST ASSISTANT BACTERIOLOGIST.

THE Minnesota State Board of Health has recently been called upon to investigate three outbreaks of an infectious disease of cattle which has proved to be of unusual interest.

The first outbreak occurred on the farm of Mr. R. W. Edmundson, near Red Rock, Minn. The first seven head of cattle were found dead, August 17, 1900. Another died August 18th. August

[2] The historical data has been collected and the clinical observations made by Dr. Brimhall; the post-mortem observations by Drs. Brimhall and Wilson; the laboratory work by Dr. Wilson, and the inoculations of and observations on vaccinated cattle by Drs. Wilson and Brimhall.

23d and August 24th four others died. August 26th another died. August 28th another, almost dead, was killed for post-mortem examination. September 1st another calf died, making fifteen animals in all from a herd of twenty-six.

The second outbreak occurred on the farm of Mr. Joseph Arth, five miles west of Forest Lake, Minn., and twenty-five north of the location of the first outbreak. Between August 21st and August 27th four head of cattle died. On August 28th, another, almost dead, was killed for post-mortem examination. On September 4th another died, making six animals out of a herd of sixteen.

The third outbreak occurred on the farm of Mr. Joseph LaVoix, one and one-half miles east of Clarissa, Minn., and one hundred and ten miles northwest of the location of the second outbreak. September 20th twelve head of cattle were found dead in the pasture. On September 24th another died. September 25th another died, and yet another almost dead was killed for post-mortem examination. September 29th another animal died, making sixteen cattle out of a herd of twenty-five.

Rumors of other outbreaks were received during the period of the above-mentioned ones, but when investigated were found to be too vague to warrant any conclusions as to their nature.

The attacks all occurred in pastures containing much brush. Some low land was present in the first instance and much in the last two. The weather during the preceding spring and early summer had been hot and dry. For several weeks previous to and throughout the period of the outbreaks the weather was hot, with frequent heavy rains. Insects of all kinds, especially mosquitoes, were very numerous. Of the thirty-seven cattle dead of the disease, four were sucking calves, twenty-eight were one to two years old, and five were three to five years old.

The symptoms, where death did not occur before the animals were seen, were fairly uniform. The animals at first appeared depressed in spirits and stiff or slightly lame when moving. The appetite was lost early, and difficulty in swallowing was present when swelling of the throat occurred. The temperature ranged from 103.6° to 105.6° F., but dropped rapidly before death. Swellings appeared most frequently on the lower portions of the legs, in the submaxillary spaces and occasionally on other portions of the body. All the animals had a black, tarry, or a bloody discharge from the bowels. Some passed bloody urine. A few had a bloody serous discharge from the nose. The animals which were sick over two days became very rapidly emaciated. Evidence of pain was present in but few cases. Most of the animals seemed to drop to the ground and die without struggling. In some of the cases death occurred within six hours after the symptoms were first observed, in most of them within twenty-four hours, while a few lived three or four days after the onset. In a total of sixty-seven animals in the three herds, thirty-seven showed symptoms of the disease, and of these none recovered, making a mortality of 100 per cent. of the affected animals. Careful disinfection with fire and corrosive sublimate, with change of well animals to new pasture, was practised in each outbreak. Quarantine measures were also rigorously maintained.

Post-mortem examinations were made of nine animals, four of which were killed when almost dead. A foamy, bloody discharge was present, post-mortem, from the nose in some cases, and clotted blood from the rectum of others. Decomposition was not unusually rapid. Abrasions of the skin of the legs were noted on some of the animals, and where these occurred the affected limbs were most swollen. On removing the skin hemorrhagic areas were found in the subcutaneous connective tissues and superficial muscles. These areas were from a pin-point in size to several inches in diameter, the proportion of large and small areas varying in the different animals. In some cases the large hemorrhagic areas were scattered over the entire body, while in others only a very few, about one inch in diameter, were found superficially. Areas showing swellings superficially were found to be hemorrhagic and œdematous to the extent of one or two inches in depth and from three to six inches in transverse diameter. The legs and submaxillary spaces contained most of these hemorrhagic and œdematous areas. The blood was slightly lighter in color, but otherwise apparently unchanged by the disease. The submaxillary glands and all the structures in the region of the larynx and pharynx in all the animals showed in greater or less degree hemorrhage and œdema. All the internal organs showed large and small hemorrhagic areas. These varied in size and numbers in the different animals. The lungs, liver, and kidneys contained the fewest large areas. No signs of purulent inflammation or fibrinous exudation were found in any of these organs. The heart muscle in most of the cases was intensely hemorrhagic. A large quantity of bloody serum was usually present in the pericardial sac. The spleen was normal in size and consistency when autopsy was made immediately after death. The color was normal except for the constant presence of a large number of hemorrhagic areas.

The stomach walls always showed several or many hemorrhagic areas involving the entire thickness of the wall. Some of these were five or six inches in diameter. The intestines always showed the same lesions as those noted in the stomach walls, and in addition in most instances there was present an intense general enteritis with bloody mucus or tarry bowel contents. In some cases there was also present a general peritonitis. The bladder walls were hemorrhagic, and in some cases a general cystitis was present, the bladder containing in these instances bloody urine.

Autopsies were made on nine of the thirty-seven animals dead of the disease. Direct cover-slip preparations, direct cultures and portions of tissue were collected from each animal from several or all of the following sources: 1. Subcutaneous hemorrhagic area. 2. Affected lymph gland (cervical or inguinal). 3. Heart's blood. 4. Lung. 5. Spleen. 6. Liver. 7. Kidney.

The direct cover-slip preparations were stained with eosin and methylene-blue. The direct cultures were made in quadruplicate on Löffler's blood serum and glucose agar and in plain and glucose broth. They were grown aërobically and anaërobically at both room and incubator temperatures. (The anaërobic cultures were made to determine the presence particularly of the bacilli of symptomatic anthrax and of malignant œdema. Cultures were kept at room temperature to aid in differentiating putrefactive organisms.) From all the animals on which autopsies were made immediately after death, namely, those designated as Red Rock No. 3, Arth Nos. 1 and 2, and La Voix No. 3, one bacillus unmixed with any other organism was obtained from all the organs tested. From all the animals on which autopsies were made several hours after death, namely, those designated as Red Rock Nos. 1 and 2, and La Voix Nos. 1 and 2, the same bacillus was obtained but mixed with (*a*) a motile bacillus with polar staining (possibly B. coli communis, possibly belonging to the same group as the special organism); (*b*) B. coli communis; (*c*) a large putrefactive bacillus growing well at room temperature and very faintly at incubator temperature; (*d*) in the spleen of Red Rock No. 2, which six days previously had been vaccinated with "black-legine," the bacilli of symptomatic anthrax were very abundant and highly virulent to guinea-pigs.

The bacillus found so uniformly present was recognized from the first as belonging to the hemorrhagic septicæmia group. It will be remembered that this group contains bacilli which are probably but varieties of the same species—shown by numerous observers to be the cause of chicken cholera, rabbit septicæmia, rinderseuche, schweinseuche (swine plague), wildseuche, buffelseuche, etc. The organism is a bacillus, though from its tendency to show marked polar staining and to form chains of shortened individuals, may be mistaken in examinations of a single specimen for a diplo- or streptococcus. The bacilli are from 0.6$\mu$ to 0.8$\mu$ in transverse diameter, and from 1.0$\mu$ to 1.5$\mu$ in length. The ends are rounded. Sometimes in cover-slip preparations from solid organs, and very frequently in those from body fluids and liquid cultures, the bacilli are in chains of three to eight individuals. In most specimens the bacilli have the ends intensely stained, and the central portion but slightly so. In some chains in rapidly growing broth-cultures this is not the case, but many of the individual bacilli are evenly stained throughout and may also be somewhat pointed at the ends. Löffler's methylene-blue brings out the polar staining to good advantage. The bacilli do not retain the stain by Gram's method. The organism is non-motile. It is aërobic, but prefers the depths rather than the surface of media. It will also give a faint growth anaërobically in glucose media even when the strictest precautions are used to exclude oxygen. It grows best at incubator temperature, and more slowly at room temperature. In plain and dextrose broth a heavy growth occurs in twenty-four hours. In Dunham's solution a small amount of indol is formed in forty-eight hours. Calpaldi Proskauer's medium is turned faintly acid after twenty-four to thirty-six hours, and partly decolorized at the bottom of the tube. Litmus milk becomes more acid and more decolorized up to forty-eight hours. No coagulation has occurred during ten days' growth in the incubator.

On Löffler's blood serum cultures direct from the diseased tissues (in every case, nine animals) do not grow well. Later cultures after several generations in the incubator in broth, etc., give a fairly abundant growth on the serum. The streaks have even edges slightly raised and are creamy white in color, not differing materially in tint from the (opaque) serum on which they are grown. On potato no growth has been obtained with any of the strains. In gelatin plates small granular white to slightly yellowish colonies appear after forty-eight hours. Those on the surface are not so large as those deeper in the medium. In gelatin stab cultures a light growth occurs on the surface, while along the needle-track numerous colonies like those in the deep portions of plate cultures develop. On plain and glycerin agar slopes a thin, narrow, granular, transparent growth occurs. The edges are slightly raised and uneven. On rabbit blood agar the growth is similar to that on plain agar, but much more abundant. In dextrose agar stab-cultures growth occurs along the entire needle-track. No gas is evolved.

The pathogenesis of the bacillus has been tested only on rabbits, guinea-pigs, and calves. Rabbits inoculated subcutaneously with small doses of pure cultures of the recently isolated organism die in from sixteen to forty-eight hours. At autopsy ecchymoses and larger hemorrhagic areas comparable in appearance and distribution to those in the cattle dead of the disease were found. The organism was present in pure culture at the site of inoculation in the heart's blood and spleen. Guinea-pigs inoculated subcutaneously with relative larger doses of the recently isolated bacilli died in from four to six days. Post-mortem examination showed a few hemorrhagic areas subcutaneously and in the internal organs. The bacilli were isolated in pure cultures from the hemorrhagic areas, heart's blood, and spleen.

It will be seen from the foregoing that the organism under investigation does not differ from the bacillus of the hemorrhagic septicæmia of other observers—with a pure culture of which it was compared in parallel series—except its tendency to form longer chains and its ability to grow slightly under anaërobic conditions.

The identity of the organism having been determined, it seemed desirable to attempt protective inoculations of exposed animals along the line of the protective inoculations against chicken-cholera already perfected by Pasteur and others. That such inoculations might be of possible good in the current outbreaks, it was decided to inoculate cattle direct without waiting to determine accurate dosage, etc., through a necessarily long series of experiments on laboratory animals. The board was able to undertake these experiments immediately through the intelligent and unselfish coöperation of Mr. Joseph Arth, on whose farm one of the outbreaks occurred. Four animals were selected for the experiments, and were designated "Vac. A," "Vac. B," "Vac. C," and "Control D," respectively. "Vac. A" was a yearling steer weighing about 400 pounds. "Vac. B" was a yearling heifer weighing about 450 pounds. "Vac. C" was a yearling heifer weighing about 550 pounds. "Control D" was a six-months-old heifer weighing about 300 pounds. The daily normal temperature of these animals ranged from 101° to 102° F.

On September 12, 1900, a mixture of twenty-four, forty-eight, and ninety-six-hour broth-cultures[1] recently isolated from cattle

[1] For the production of resistance it is probable that older cultures should have been employed.

dead of the disease on the Arth farm were filtered through porcelain and the filtrate used for inoculation subcutaneously of the first three animals. "Vac. A" was given 1.5 c.c., "Vac. B" 1 c.c., and "Vac. C" 0.75 c.c. The temperature of all rose slightly within the next twenty-four hours, that of "Vac. A" rising 1.5° F. "Vac. A" also showed a slight swelling and soreness at the site of inoculation. The animals remained in good health for the next eight days.

On September 20th a mixture of equal parts of a twenty-four-hour and a forty-eight-hour broth-culture of the bacillus recently inoculated from another animal dead on the Arth farm was shaken up with an excess of chloroform and allowed to stand for three hours in a tightly stoppered flask. The stopper was then removed, the flask plugged loosely with sterilized cotton and heated for one hour to 60° C. The supernatant fluid was then decanted from the sediment and used for subcutaneous inoculation of the first three animals of the series. Each animal was given 5 c.c. The temperatures of all were but slightly elevated at the end of the next twenty-four hours. "Vac. A" was then given 10 c.c. more of the heated culture noted above (which in the meantime had stood for twenty-four hours in a warmly-heated room, giving opportunity for the multiplication of any bacteria not killed by the previous treatment). Within twelve hours the animal's temperature had risen 3° F. It was apparently quite sick from this on, though no swelling occurred except at the site of inoculation, and no bloody discharges were present. The animal died thirty-seven hours after the last inoculation.

Post-mortem examination showed a very large hemorrhagic area at the site of the last inoculation. None was present at the sites of the first and second inoculations. Small hemorrhages were also present elsewhere in the subcutaneous tissues and internal organs, though not so abundant nor so large as those seen in the majority of the animals dead of the disease through natural infection. The legs were affected as in the other animals.

Direct cover-slip preparations and cultures from the site of inoculation, spleen, and heart's blood gave the bacillus inoculated in pure culture.

The question might fairly be raised whether the animal was killed by a natural infection, by too large a dose in the experimental inoculation, or by a combination of the two. Unfortunately there is no way of determining the matter absolutely, since the opportunity of studying the exact nature of the material used for the third inoculation was lost at the time.

On October 2d a mixture of a twenty-four-hour, forty-eight-hour, and a twelve-day culture from an animal dead of the disease on the Arth farm was used for inoculating subcutaneously animals "Vac. B" and "Control D." "Vac. B" was given subcutaneously 25 c.c. of the mixture, and "Control D" 10 c.c. of the mixture.

The roan heifer, "Vac. C," was also placed under the same surroundings as to food, housing, etc., and her temperature taken as control for the two inoculated animals. The temperatures for the next sixty hours were as follows:

| | Vac. B. | Control D. | Vac. C. |
|---|---|---|---|
| Animals just driven in from pasture: | | | |
| October 2d, 5 P.M. . . . . | 103.3° | 103.6° | 103.4° |
| Animals inoculated as noted above: | | | |
| October 2d, 9 P.M . . . . | 102.9° | 105.3° | 102.4° |
| " 3d, 6 A.M. . . . . | 102° | 103.8° | 101° |
| " 3d, 12 M. . . . . | 102° | 103.9° | 102° |
| " 3d, 6 P.M. . . . . | 102.4° | 104.2° | 102° |
| " 4th, 6 A.M. . . . . | 101.2° | 104.4° | 101.2° |
| " 4th, 12 M. . . . . | 102° | 104.3° | 102.1° |
| " 4th, 6 P.M. . . . . | 102.6° | 104.8° | 102.4° |
| " 5th, 4 A.M. . . . . | 102° | 105.4° | 101.3° |

The calves were all driven in from pasture at the same time and placed in stanchions in a dry barn. They were fed hay only during the observations. The weather was very warm for the season of the year, and moist. The animal previously inoculated, designated "Vac. B," showed absolutely no symptoms from the inoculation of the live culture except a slight swelling and tenderness at site of inoculation for the first thirty-six hours. The animal previously uninoculated, designated "Control D," showed a marked rise of temperature (see table), which remained permanently elevated. The animal had a marked swelling and tenderness at site of inoculation which extended to the adjacent regions. Lameness was present after the first twelve hours. The animal remained lying on its side with its forelegs drawn up in a constrained position. At the end of the first twenty-four hours all the observers were positive the calf would be dead before morning, but while still very sick the next morning, after thirty-six hours, the calf was not dead, and by 8 A.M., thirty-eight hours after inoculation, was apparently feeling somewhat better. At this time, in order to if possible hurry up the experiment and kill the animal more quickly, it was given subcutaneously on the left side of the abdomen 15 c.c. more of the mixture used for the original inoculations. The symptoms were intensified from this on.

On October 11, 1900, "Control D," which since its last inoculation, October 5th, had appeared to be sick, though able to eat and move about fairly well, was killed and an autopsy made at once. No evidence of affection of subcutaneous tissue about the ankles was visible. On removing the skin at the sites of inoculations there was found a small cavity (several inches in diameter) surrounded by a large area very œdematous and showing old and more recent hemorrhages. At one site of inoculation were two separate cavities, one very markedly circumscribed and filled with a clear fluid resembling serum more than pus. The other corresponding to this was not sharply defined, but seemed to be composed of pockets lying in the interstices of the tissue. At some distance from one œdematous area there were found a few very small hemorrhagic areas exactly resembling those seen in the clinical cases.

The lymph glands in front of the shoulder and along the neck generally—*i. e.*, those draining the area directly affected by the inoculation—were all enlarged. The smaller ones were black and hemorrhagic throughout, and the larger ones were much darker than normal, apparently in a state of partial recovery from previous general hemorrhagic infiltration, and contained here and there a few recent hemorrhagic areas.

The inguinal glands were similarly affected. No pathological lesions were found in any of the internal organs except the kidneys, which were much swollen, with old and recent hemorrhages between the capsule, in the pelvis, and between the lobules.

Direct cover-slip preparations, cultures and tissues from (*a*) pus cavity at site of inoculation, (*b*) œdematous area surrounding site of inoculation, (*c*) involved lymph glands, (*d*) spleen, (*e*) kidney were collected. The kidneys were preserved in Kaiserling's fluid.

After ninety-six hours in the incubator all cultures except those from pus at site of inoculation remained sterile. Cultures from pus at site of inoculation gave pure cultures of the inoculated organism.

SUMMARY. 1. Three outbreaks of hemorrhagic septicæmia in cattle—hitherto, it is believed, not reported as occurring in these animals in the Northwest—have occurred in Minnesota during August and September, 1900.

2. Of sixty-seven animals in the three herds, thirty-seven showed symptoms of the disease, and all such died—a mortality of 100 per cent. of the affected animals.

3. The chief symptoms were loss of appetite, fever, stiffness, swelling of legs and submaxillary region, and a black, tarry, or bloody discharge from the bowels. Bloody urine and bloody nasal discharge were present in some cases. Death occurred usually in from six to twenty-four hours after the first appearance of the symptoms.

4. The chief lesions discovered at autopsy were ecchymoses and large hemorrhagic areas in the subcutaneous connective tissue muscles, lymph glands, and throughout all the internal organs. The cervical lymph glands, heart muscle, spleen, and alimentary canal were most affected.

5. From the nine animals on which autopsies were made the same bacillus was obtained from all tissues examined. Where the examination was made immediately after death—four cases—it was unmixed with any other organism.

6. The bacillus was identified as belonging to the group causing chicken cholera, rabbit septicæmia, rinderseuche, schweinseuche (swine plague), wildseuche, buffelseuche, etc., by direct cover-slip preparations, parallel cultures in various media, and by inoculation of animals in which the characteristic lesions were thus reproduced and from the tissues of which the inoculated bacilli were recovered in pure culture.

7. An attempt was made to immunize cattle by the injection of filtered, and, later, of killed cultures of the bacillus. The experiments are as yet too few and the results not sufficiently tested to warrant conclusive statements as to the protective value of the inoculation, but it would appear that a fairly high degree of immunity has thus been produced.

